# Beneficial and adverse effects of vitamin E on the kidney

**DOI:** 10.3389/fphys.2023.1145216

**Published:** 2023-03-15

**Authors:** Aldona Baltusnikiene, Inga Staneviciene, Eugène Jansen

**Affiliations:** ^1^ Department of Biochemistry, Lithuanian University of Health Sciences, Kaunas, Lithuania; ^2^ Retired from Centre for Health Protection, National Institute for Public Health and the Environment, Bilthoven, Netherlands

**Keywords:** vitamin E, kidney, biomarkers, upper limit of toxicity, supplementation, oxidative stress

## Abstract

This article reviews the beneficial and adverse effects of high-dose vitamin E supplementation on the vitamin E status and renal function in human and rodent studies. The high doses of vitamin E, which can cause renal effects, were compared to upper limits of toxicity (UL) as established by various authorities worldwide. In recent mice studies with higher doses of vitamin E, several biomarkers of tissue toxicity and inflammation were found to be significantly elevated. In these biomarker studies, the severity of inflammation and the increased levels of the biomarkers are discussed together with the need to re-evaluate ULs, given the toxic effects of vitamin E on the kidney and emphasizing oxidative stress and inflammation. The controversy in the literature about vitamin E effects on the kidney is mainly caused by the dose-effects relations that do not give a clear view, neither in human nor animals studies. In addition, more recent studies on rodents with new biomarkers of oxidative stress and inflammation give new insights into possible mechanisms. In this review, the controversy is shown and an advice given on the vitamin E supplementation for renal health.

## 1 Introduction

Vitamin E is an essential micronutrient and a fat-soluble vitamin that acts as an antioxidant in the detoxification chain of fat-soluble to water-soluble toxic radicals. Therefore, vitamin E plays an important role protecting various tissues from lipid peroxidation. Furthermore, vitamin E has also several protein dependent functions and gene modulation effects in inflammation.

**FIGURE 1:**
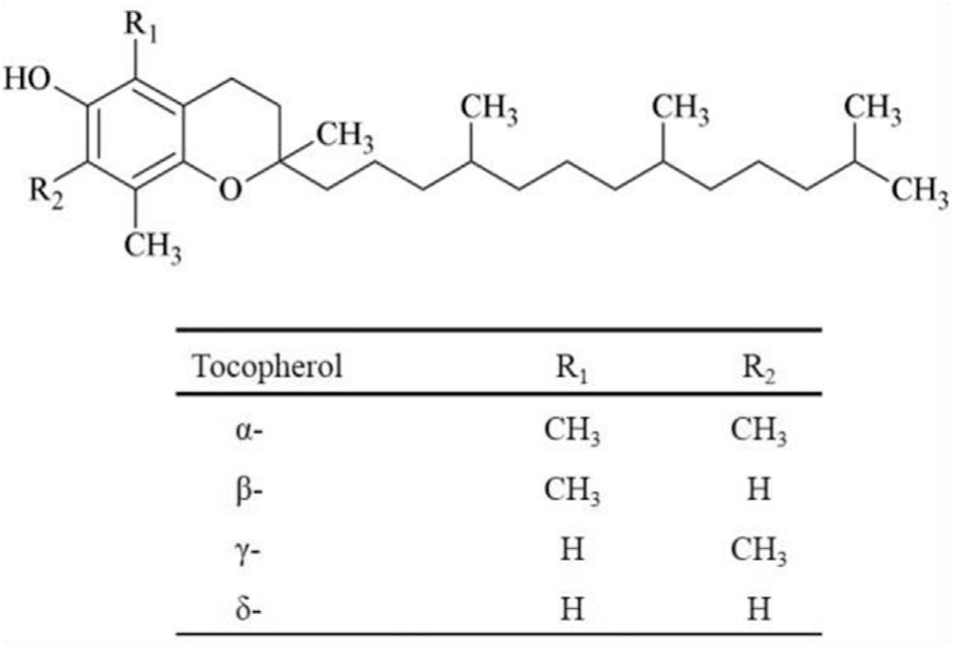
Chemical structures of the four tocopherols.

The vitamin E family contains four naturally occurring structurally similar substances, saturated homologs, α-, β-, γ-, and δ-tocopherols. Chemical structures of tocopherols are shown in Figure 1 (Ungurianu et al., 2021).

The chemical structure of vitamin E includes a chromanol ring with one hydroxyl group and three, two or one methyl group on the aromatic ring. As a result, α-tocopherol with three methyl groups is the most active of the tocopherols and δ-tocopherol with one methyl group is the least active (Ungurianu et al., 2021). Each form has slightly different biological activities (Burton and Ingold, 1981).

**FIGURE 2:**
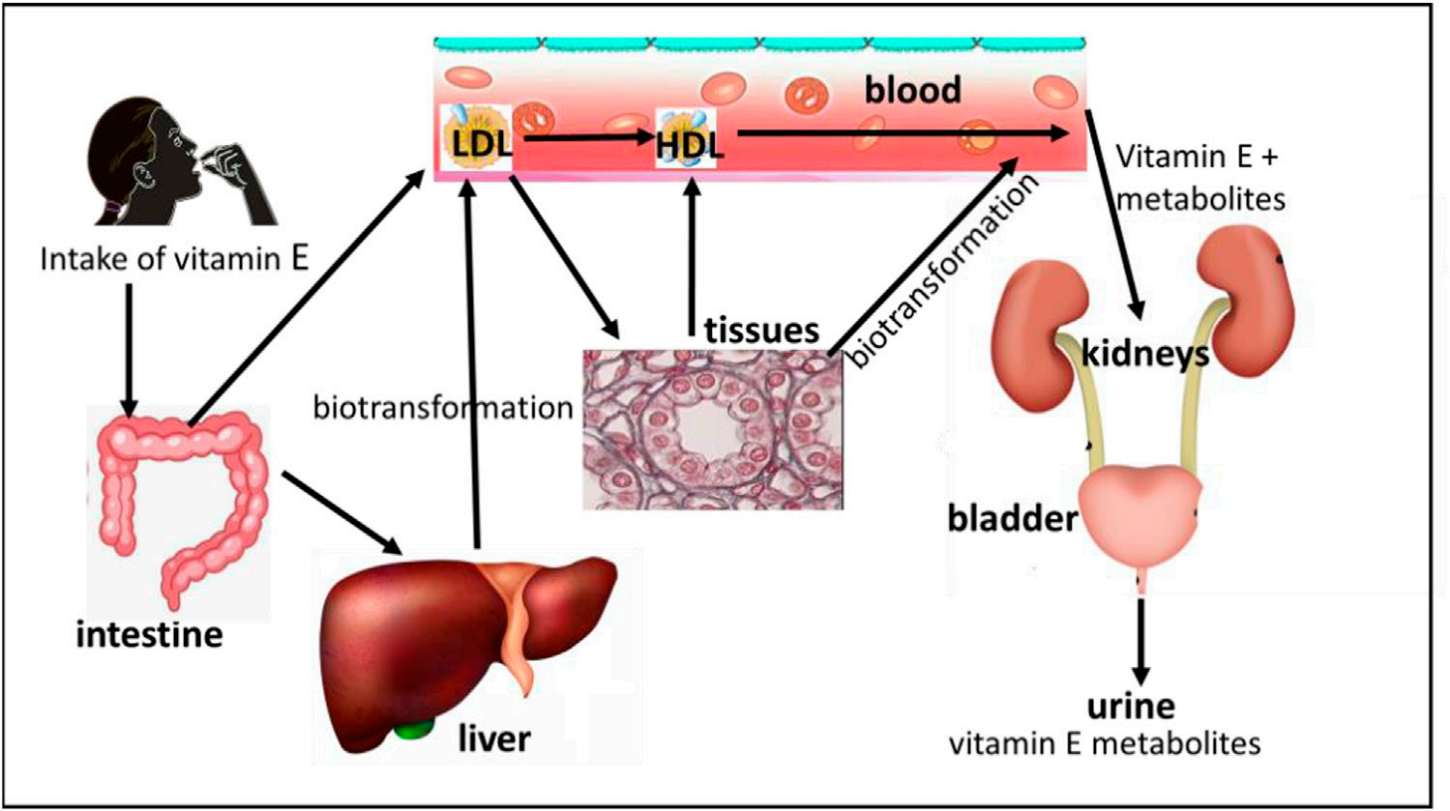
A schematic representation of the metabolic fate of vitamin E.

**FIGURE 3:**
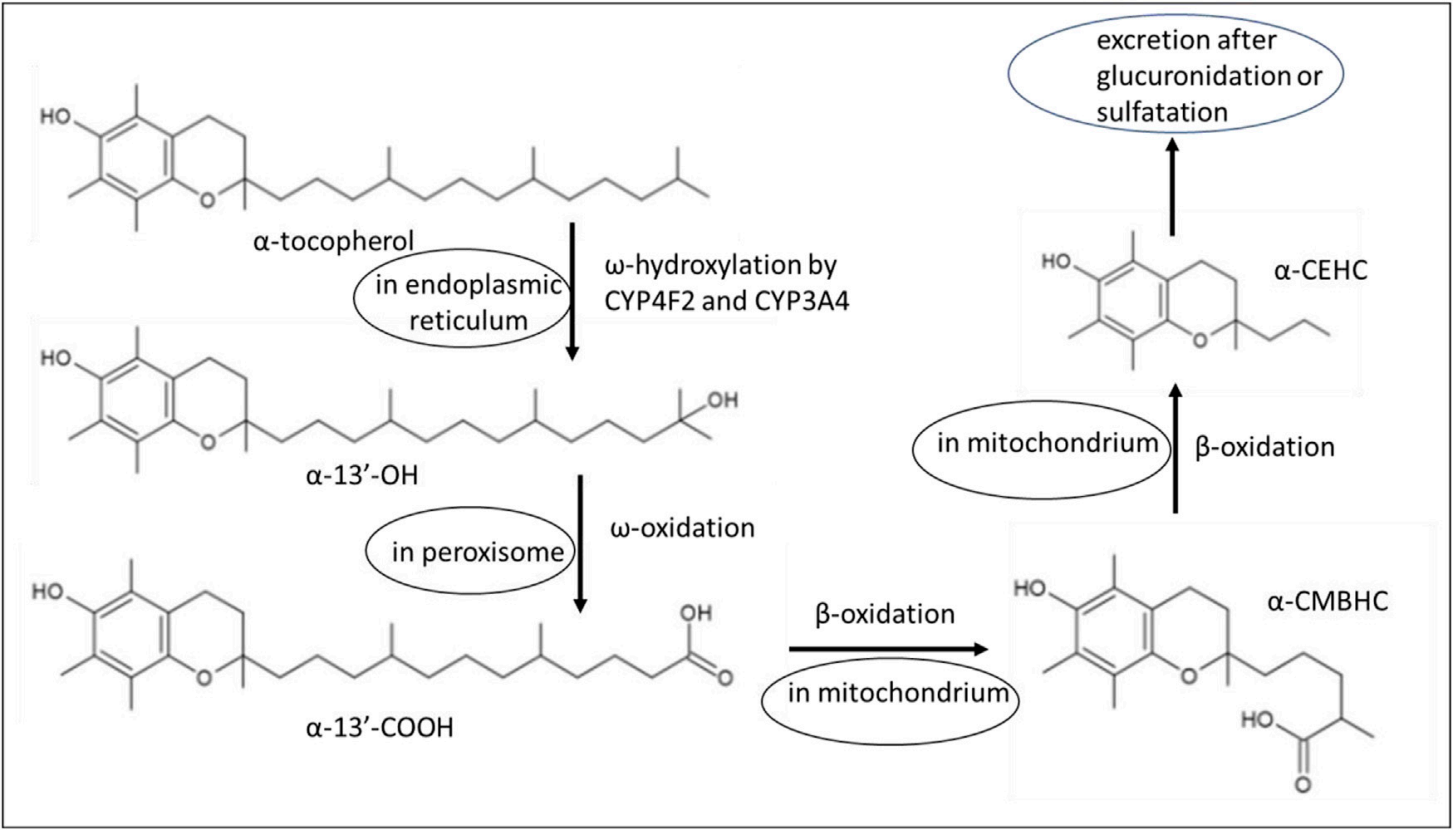
A detailed scheme with chemical structures of α-tocopherol metabolites in various cellular departments. Explanation of the chemical structures: α-13′-OH, 13′-hydroxychromanol; α-13′-COOH, 13′-carboxychromanol; α-CEHC, carboxymethyl butyl hydroxy chromanol; α-CMBHC, carboxymethyl hexyl hydroxy chromanol.

Vitamin E metabolism involves liver binding proteins that selectively bind nutritional α-tocopherol. The specific α-tocopherol transfer protein mediates its transport from the liver to lipoproteins, mainly LDL but also HDL and VLDL (Brigelius-Flohé, 2007). The primary function of the tocopherol transfer protein is to maintain homeostatic control of α-tocopherol concentrations in blood and other tissues, and it is also expressed in the kidney (Herrera et al., 2001). A simplified schematic representation of the metabolic fate of vitamin E is shown in Figure 2. A detailed metabolic scheme of α-tocopherol in the various cellular departments, endoplasmic reticulum, peroxisomes, and mitochondrion, is depicted in Figure 3.

Vitamin E plays an important role in the kidneys partially due to its antioxidant properties. The most important disorder of the kidney is the so-called chronic kidney disease (CKD). This disease affects >10% of the general population and also shows an increase in associated deaths over the last two decades (Kovesdy, 2022).

CKD can be defined as a condition of relative deficiency and impaired metabolism of vitamin E (Rojo-Trejo et al., 2022). CKD is also characterized by an increased state of oxidative stress. It is argued that oxidative stress is the main contributing factor to the high cardiovascular morbidity and mortality in CKD patients. Moreover, CKD is also related to high inflammation, which results in a pro-oxidative situation. Vitamin E deficiency is often found in patients with CKD, which is associated with multiple side effects in the kidney (Galli et al., 2022). In a recent comprehensive review of Galli et al. (2022), an overview is presented of the roles of vitamin E in the kidney. Not only its antioxidant properties are important, but also the alterations of the enzymatic vitamin E metabolism as found in patients with CKD, resulting in large changes in vitamin E metabolites concentrations in plasma. In addition, the altered metabolism is associated with an increase of the ω-6/ω-3 ratio of long chain fatty acids, resulting in a pro-inflammatory phenotype in patients with CKD. However, a higher intake of α-tocopherol did not lead to a significant effect reduction of mortality in patients with CKD. Clearly, the vitamin E metabolism and its effect on the kidney are very complicated.

Potential benefits and disadvantages of vitamin E have been described in many animal models and human clinical trials. In this review, we try to explain these contrary effects of vitamin E and relate this to available supplementation, the upper limits of toxicity, gender effect and finally try to come to an advice for the vitamin E supplementation under various conditions.

## 2 Intake of vitamin E

### 2.1 Dietary intake of vitamin E

There are two ways to express the dose and the amount of fat-soluble vitamins in a product: one way is based on quantity, where the unit of measurement is mg/day and the other way is based on biological activity, where the unit of measurement is an international unit (IU). A supplement of α-tocopherol 400 IU/day is equivalent to 268 mg/day. In this report we often use the term vitamin E when we mean α-tocopherol.

**FIGURE 4:**
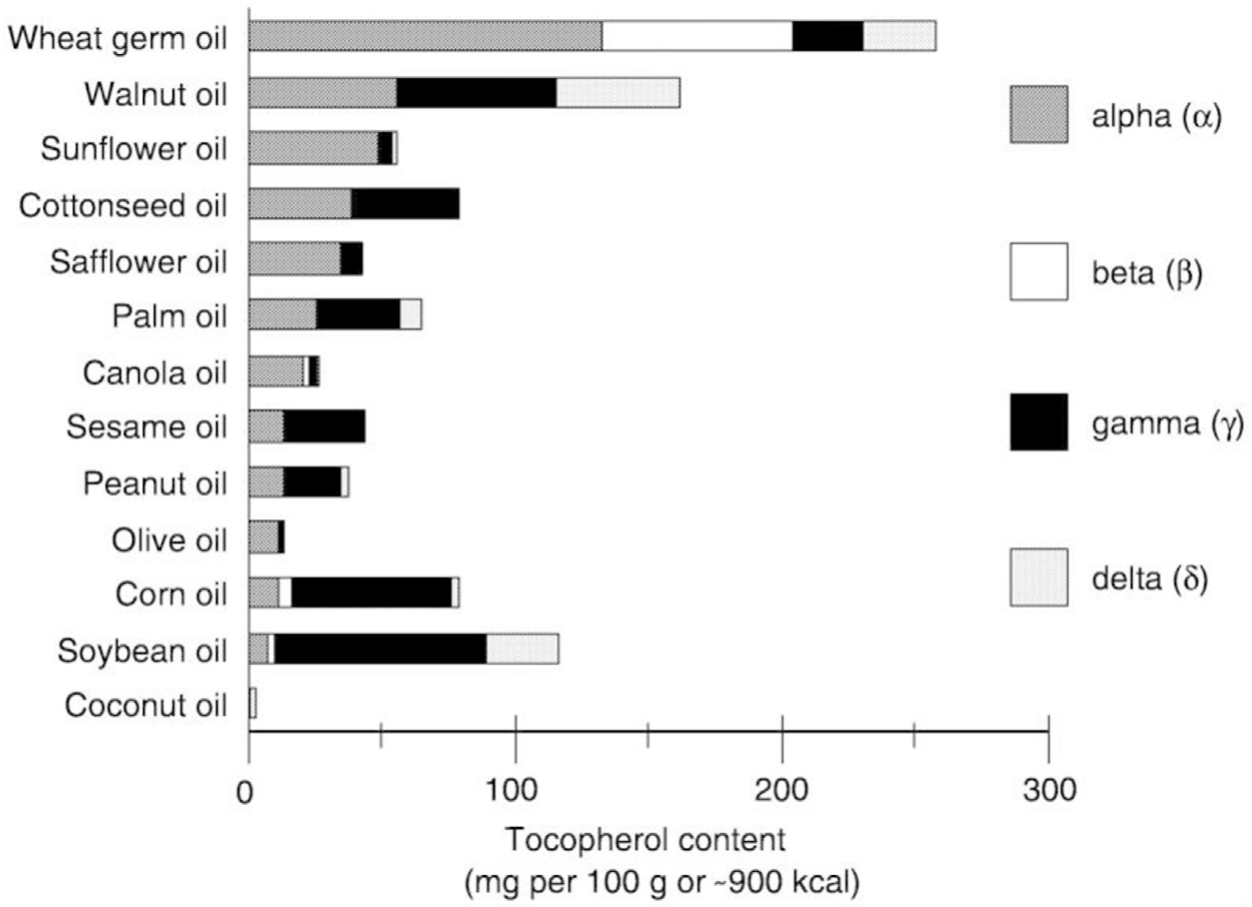
Content of the four homologues of vitamin E in oils. (From: NCBI, NBK225461).

Foods rich in vitamin E are mainly edible oils and nuts, such as wheat germ oil, sunflower oil, hazelnut oil, almond oil and nuts such as almonds, hazelnuts, pine nuts, peanuts, Brazil nuts, etc. The different fats and oils contain a rather different pattern of the four homologs of vitamin E. Because α-tocopherol is the most abundant form in edible oils, α-tocopherol is responsible for almost 90% of vitamin E activity in human tissues. The relative potency of α-, β-, γ- and δ-tocopherols is reported to be approximately 100:38:9:2 (Manolescu et al., 2008). A comprehensive overview of the contents in food of homologs of vitamin E can be found in a book chapter by Niki and Abe (2019), also for plants, fish and other animals. Figure 4 shows an overview of the content of tocopherols in oils.

The recommendation of most food authorities to maintain adequate vitamin E status is about 12–15 mg of vitamin E per day. Because vitamin E is present in sufficient amounts in the Western diet, deficiencies will not occur to a large extent. In a comparative study by Beulens et al. (2010), the intake of vitamin E derived from questionnaires was determined in the MORGEN study of 10,260 men and 12,394 women. The calculated intake was 15.2 and 12.6 mg/day for men and women, respectively. For adults, the recommended intake for vitamin E has been set by the US FDA 13 mg/day for men and 11 mg/day for women (Food and Drug Administration, 2016).

While there is some variation in dietary intake of vitamin E, serum levels are quite similar between individuals and also between countries. Cook-Mills and McCary (2010) and Wagner et al. (2004) made an inventory which showed that the mean variation between 12 studies in Western countries was 21–27 μmol/L. However, blood concentration of vitamin E is not always a good indication of vitamin E status. This is due to the strong homeostasis of vitamin E, which keeps the blood concentration constant on account of vitamin E in the tissues. Therefore, it is important to get enough vitamin E to maintain tissue concentrations, regardless of blood status.

### 2.2 Intake of vitamin E through supplementation

It is clear from the above that with a normal diet it is not possible to have too high an intake of vitamin E to cause adverse effects. But as for high doses of vitamin E, obtained from the Internet or local stores, it is a different story. The authorities in Europe (EFSA) and Australia/New Zealand (Australian National Health and Medical Research Council and the New Zealand Department of Health) have set an upper limit for vitamin E toxicity of 300 mg/day. On the Internet it is possible to buy vitamin E in doses of 268 mg/day plus 44 mg of a natural tocopherol mixture. Thus achieved 312 mg intake of a vitamin E blend exceeds the UL of 300 mg/day (Orthica E-400 + mini soft gels).

The highest dose we have found available on the Internet is from Solgar Vitamins. They sell vitamin E in daily doses of 670 mg of (1000 IU, 5,592% of the RDI) plus 44 mg of other tocopherols, so a total intake of 715 mg of tocopherols.

Although vitamin E is a common term for a mixture rich in tocopherols, several variations in composition and formulation are available on the internet. Some examples are given in Table 1. The information about the content of different tocopherol homologs is not always given.

**TABLE 1:** Summary of available vitamin E formulations and vitamin E composition.

Companies	Vitamin E amount	Composition of vitamin E
Now Foods	268 mg	α-tocopherol
Orthica	268 mg	α-tocopherol
Swanson	268 mg	α-tocopherol
Green Day	268 mg	α tocopheryl acetate
Nature’s Bounty	450 mg	α-tocopheryl acetate
Now Foods	670 mg	mixed tocopherols (α, β, δ, γ)
Solgar	670 + 44 mg	α-tocopherol + β-, δ- and γ-tocopherol

Because the high doses of vitamin E are also available in Europe, the European Commission has started in 2002 an initiative law to harmonize and limit the maximum content of vitamins and minerals in supplements to 1x the recommended daily intake, for vitamin E it would be 15 mg. But what happened to this proposal? In a Regulatory Affairs Report from 2018, Coppens states: “After the adoption of the Food Supplements Directive 2002/46, the Commission published a report in 2008 on further harmonization in this area” (Commission of the European Communities, 2008). He concluded that harmonization would be difficult to achieve given the different positions of the Member States and the scientific issues that must first be resolved (Coppens, 2018). Member States with undefined maximum levels feared that EU harmonization would limit their market (Government of the UK, Expert Group on Vitamins and Minerals, 2003). Commercial pressure would probably also be one of the reasons.

## 3 The upper limits of toxicity of vitamin E for humans

The upper limit of toxicity (UL) for vitamin E has been set by several leading authorities worldwide.

### 3.1 Europe (European Union)

In Europe, the European Food Safety Authority (EFSA) has derived a UL for different age groups, the so-called tolerable upper limit of intake (EFSA, 2015). For adults, this UL was set at 300 mg of vitamin E per day. In their evaluation, EFSA concluded that several biochemical indices, such as the metabolism of iodine, did not have enough toxicological significance to use these data. Blood coagulation was considered the most important effect to determine UL, as described in a study by Meydani et al. (1998). In this study, the NOAEL was 540 mg/day and EFSA concluded that an uncertainty factor of two sufficiently covered inter-individual differences in sensitivity. The UL for vitamin E was therefore set at 270 mg/day for adults (≥18 years) and rounded to 300 mg/day.

### 3.2 United States of America (National Health Institutes)

The upper limit of the Tolerable Intake (UL) in the US is 1,000 mg/day, equivalent to 1500 IU of vitamin E. This UL of 1,000 mg of vitamin E/day was based on the adverse effect of increased bleeding observed in rat studies (Wheldon et al., 1983; National Academies of Sciences, 2000). The tolerable upper intake limits (ULs) for vitamin E were not different for men and women and were the same during pregnancy and during lactation in the same age groups. Hatchcock et al. (2005) confirmed the UL of 1,000 mg/day in a detailed analysis. They stated that there is no strong evidence of possible adverse effects of high vitamin E intake in humans. Therefore, they concluded that vitamin E supplements appear to be safe for most adults in amounts ≤1600 IU (1,073 mg/day).

Attempts have been made in scientific debates to answer questions about adverse effects of vitamin E by combining the results of multiple studies. An analysis of data from 19 clinical trials of vitamin E found a higher mortality rate in trials where patients took more than 400 IU (=268 vitamin E/day) *via* supplementation. But there are limitations to these conclusion, as other meta-analyses have come to different conclusion (Miller et al., 2005).

### 3.3 England (National health services)

The National Health Service in England stated that 540 mg/day (800 IU) or less of vitamin E supplements are unlikely to do any harm (National Health Services of the UK, Government of the UK, 2003).

The Ministry of Health, Welfare and Sport advised that it should be possible to get the required amount of vitamin E by eating a varied and balanced diet. And if vitamin E supplements are taken, it should be done in low quantities, as it can be harmful. Taking vitamin E supplements at 540 mg/day or less is unlikely to cause any harm (Last Revised: 03 August 2020). The level of 540 mg/day was derived from a dose-dependent study in 88 healthy volunteers (>65 years, over 4 months. In the evaluation, the UK authorities did not use an uncertainty factor of two for inter-individual differences in sensitivity as EFSA did. Meydani et al. (1998) focused on a wide range of possible adverse effects. They found no effect on many biomarkers in serum/plasma. The only renal-related biomarkers were urine and serum creatinine that also were no affected by vitamin E exposure.

### 3.4 Australia and New Zealand

https://www.nrv.gov.au/nutrients/vitamin-e.

The Australian National Health and Medical Research Council (NHMRC) and the New Zealand Department of Health (MoH) have set a UL for vitamin E of 300 mg/day. This UL was based on the study by Meydani et al. (1998) and used the same arguments with a safety factor of two and a rounding of 270–300 mg/day as the EFSA did (Nutrient Reference Values for Nutrient Reference Values, 2005).

### 3.5 Other stake holders

The Health Food Manufacturers’ Association in August 2011 revised the permitted vitamin E level to a maximum of 675 mg (1000 IU) which can be supplied accompanied by label advice stating “not intended for long-term use” or “for use under coaching from a practitioner” (HFMA, 2018).

Table 2 summarizes the ULs of four leading global authorities.

**TABLE 2:** Summary of ULs for adults (19+ yrs.), expressed as vitamin E (mg/day), determined by various worldwide authorities.

Country/Continent	Authority	UL for vitamin E (mg)
Europe	EFSA	300
Australia + New Zealand	NHMRC	300
Great Brittany	NHS	540
United States	NIH	1,000

For pregnancy and lactation, the same ULs were taken as for adults.

In the following chapters we focus on the comparison of the doses causing potential adverse effects in human and rodent studies with the ULs.

## 4 Benefits of vitamin E for the kidneys in human studies

A number of experimental and clinical studies in humans have shown that vitamin E may play a role in preventing or ameliorating kidney damage.

Several other studies also indicated an association between dietary intake of vitamin E, as measured by food questionnaires, and improved kidney function. A study conducted in Iran found that a higher dietary intake of vitamin E was associated with a lower risk of incident CKD (Farhadnejad et al., 2016).

There are more studies with patients with kidney disease reporting beneficial effects of vitamin E through diet or supplementation. Boaz et al. (2000) investigated the effect of a high dose of vitamin E of 800 IU/day (= 536 mg) in haemodialysis patients with pre-existing cardiovascular disease for 159 days. Vitamin E supplementation reduced several endpoints of cardiovascular disease and myocardial infarction.

In other intervention studies, vitamin E supplementation reduced the risk of acute kidney injury in CKD patients and improved renal function in diabetic patients, as reviewed by Di Vincenzo et al. (2019). Mune et al. (2018) reported a study in which patients with end-stage renal disease on haemodialysis received vitamin E (300 mg per day) for 12 weeks. Vitamin E improved HDL function of cholesterol outflow capacity and also endothelial function. Bolignano et al., (2017), however, concluded that there is no solid evidence for the benefits of antioxidant supplementation of vitamin E for slowing the progression of diabetic kidney disease. In patients with diabetic kidney disease, antioxidants may improve early kidney damage. Aghadavod et al. (2018) showed that high-dose vitamin E supplementation for 12 weeks had beneficial effects on the lipid profile and glutathione levels of 54 patients with diabetic nephropathy. Bursell et al. (1999) conducted a 4-month study in type 1 DM patients who received 1,800 IU of vitamin E/day (1,206 mg/day). Vitamin E treatment appeared to normalize renal hemodynamic abnormalities and improve renal function, thereby reducing the risks of developing nephropathy.

Khatami et al. (2016) described a trial with patients with diabetic nephropathy taking 1200 IU of vitamin E/day (804 mg/day). The vitamin E intervention showed that high-dose vitamin E supplementation for 12 weeks in patients with diabetic nephropathy had beneficial effects on biomarkers of kidney damage, inflammation (TNF-α), oxidative stress and insulin concentrations.

However, there are also some conflicting results on the effects of vitamin E intake and renal function (Di Vincenzo et al., 2019). In a study of young adults, Hirahatake et al. (2019) found no association between vitamin E serum concentrations and eGFR decrease.

From a meta-analysis (Abner et al., 2011), vitamin E supplementation appears to have no effect on all-cause mortality at doses up to 5,500 IU/day (=3,850 mg/day).

Beneficial effects of vitamin E supplementation for subjects with kidney diseases are controversial. The HOPE study found no beneficial effects of vitamin E supplementation of 268 mg/day on CVD mortality or even renal complications (Mann et al., 2004). In a study by Meydani et al. (1998) that provided the baseline information for the ULs for vitamin E supplementation, they focused on several possible adverse effects. The only renal-related biomarkers were urinary and serum creatinine, which also showed no effect from vitamin E exposure at a dose of 540 mg/day.

In conclusion, it is difficult to make a unified statement about the benefits of vitamin E in human kidneys. Reports by Mann et al. (2004) and Miller et al. (2005) concluded that vitamin E supplementation in CKD patients is not recommended, as evidenced by its negligible effect on all-cause mortality. From the result of many studies, it can be concluded that vitamin E intake through normal diet or supplementation at a low level has certain benefits for patients with CKD and HD. However, the diversity and heterogeneity of the research population must be considered, such as age, gender, ethnicity and any comorbidities. Thus, further studies are needed to investigate whether there is a threshold for the amount of vitamin E supplementation that still has beneficial effects. Conflicting results have been published on the beneficial or adverse effects of vitamin E supplementation, which depend on the doses used. Galli and Azzi, (2010) stated in a meta-analysis that CKD can be considered as a condition of relative deficiency and impaired metabolism of vitamin E, possibly due to increased oxidative stress.

## 5 Adversary effects of vitamin E on the kidneys in human studies

In addition to the beneficial effect of vitamin E supplementation, adverse effects of high doses have hardly been reported.

In chronic kidney disease (CKD) studies, there is too little information and also discussion about the effects of vitamin E levels or vitamin E supplementation. CKD patients have plasma vitamin E levels that are usually within a normal range, although reduced vitamin E concentration was observed in erythrocyte membranes of CKD patients (Rojo-Trejo et al., 2022). Plasma vitamin E levels were also significantly lower with renal disease severity. These findings confirm other studies that reported significantly reduced vitamin E levels in advanced stages of CKD patients (Montazerifar et al., 2010).

The therapeutic and beneficial effects of vitamin E are evidenced by its increased antioxidant status as well as some other cellular processes. But there is also concern about the potential adverse effects of vitamin E, particularly with the availability of large supplemental doses, higher than the UL as determined by EFSA, in England and Australia, but still lower than the UL for the US. Several authors have shown that high doses of vitamin E can also interfere with drug metabolism (Galli et al., 2017).

In the HOPE study, supplementation of 400 IU (=268 mg) of vitamin E/day in mild to moderate CKD patients has no effect on cardiovascular outcomes and overall mortality and therefore supplementation of vitamin E is not recommended (HOPE Study Investigators, 2000). Vitamin E has been shown to be safe in varying amounts over a four-month period in healthy elderly subjects (Meydani et al., 1994; Meydani et al., 1998). Vitamin E was administered at 60, 200, or 800 IU for 4 months. A number of tests assessed health, nutritional status, and the function of the liver, thyroid, and kidneys. No adverse effects were observed in this older group after vitamin E supplementation. Therefore, it was concluded that this intake of vitamin E is safe. In a recent human study of haemodialysis patients with pre-existing cardiovascular disease, a vitamin E dose of 800 IU/day of vitamin E (=540 mg/day) was administered for 519 days. Treatment with vitamin E reduced the risk of myocardial infarction in patients with coronary atherosclerosis (Boaz et al., 2000; Sozen et al., 2019).

There is still no convincing evidence that vitamin E supplementation is beneficial as a therapy for slowing down CKD. A possible change in the recommendations can only be expected after future studies with a longer follow-up and a larger sample size. Preclinical studies showed that increased vitamin E concentrations caused a significant increase in cytochrome P-450-3A protein expression. Since CYP3A4 is involved in the metabolism of many drugs, it is likely that high doses of vitamin E would affect the metabolism of a large number of drugs (Ungurianu et al., 2021).

In human studies, the findings of serious adverse effects of vitamin E supplementation on the kidneys are very rare. As shown in Table 3, only the beneficial and no-effect scores were found, although in five studies vitamin E supplementation was above the UL of 300 mg/day and in one study, vitamin E supplementation was even above the UL of 1,000 mg/day.

**TABLE 3:** Summary of human studies that reported effects of vitamin E supplementation on the kidneys.

Author	Dose (mg/day)	Overall effect	Participants	Number of participants	Duration	Country	Detailed effects
Himmelfarb et al. (2003)	200	+	Haemodialysis patients	30	14 days	United States	Anti-inflammatory and anti-oxidative effects
Mann et al. (2004)	268	0	Patients with renal insufficiency	9,541	4.5 years	19 countries	No effects on cardiovascular outcomes
Mune et al. (2018)	300	+	Diabetic and non-diabetic patients with ESRD	40	12 weeks	Japan	Amelioration of endothelial function in diabetic subjects
Boaz et al. (2000)	536	+	Haemodialysis patients with CV disease	196	519 days	Israel	Reduction of CV disease endpoints and myocardial infarction
Meydani et al. (1998)	727	0	Healthy elderly	88	4 months	United States	No adverse effects on renal functions
Khatami et al. (2016)	804	+	Type 2 diabetes with kidney disease	60	3 months	Iran	Reduction of proteinuria, oxidative and inflammatory markers
Giannini et al. (2007)	804	+	Type 1 diabetes with renal disease	10	12 months	Italy	Positive effects on oxidative stress
Bursell et al. (1999)	1,206	+	Type 1 diabetes patients	45	8 months	United States	Normalize retinal hemodynamic abnormalities and improve renal function

+, overall positive effects; 0, overall no significant effects.

## 6 Beneficial effects of vitamin E for the kidneys in rodent studies

Several animal models have shown beneficial effects of vitamin E administration. A review by Rojo-Trejo et al. (2022) discusses several rodent studies that reported beneficial effects of vitamin E supplementation, including in the kidneys. In a study by Koya et al. (1997) it was shown that vitamin E can play a role in the prevention of diabetic nephropathy with vitamin E supplementation of 40 mg/bw/day by intraperitoneal injection.

Zhao et al. (2018) conducted a study in rats, including a diabetic group, with a dose of 1,000 mg/kg bw of vitamin E *via* oral gavage for 12 and 24 weeks. Vitamin E was shown to improve all clinical biomarkers in the kidney in diabetic rats. In addition, vitamin E treatment attenuated cellular apoptosis and interstitial fibrosis in the renal cortex, while normal rats showed no effect on vitamin E treatment.

In dietary treatment with vitamin E in rats with increased kidney damage from oxidants, vitamin E can attenuate these effects. In this study by Trachtman et al. (1996) rats were fed a diet of 100 IU (67 mg) of vitamin E/kg of feed. In another study using the same supplementation of vitamin E, Trachtman et al. (1995) also showed beneficial effects on kidney structure and function associated with decreased levels of malondialdehyde in the kidneys and liver. In a rat study by Hahn et al. (1998) vitamin E has the potential to modulate both tubulointerstitial injury and glomerulosclerosis, inhibit TGF-β and decrease renal malondialdehyde concentration.

## 7 Adverse effects of vitamin E on the kidneys in rodent studies

Sub chronic toxicity in rats was achieved by administering alpha-tocopheryl acetate by gavage at a dose of approximately 500 mg/kg body weight/day. Biochemical indices of hepatotoxicity and CKD being serum ALT and AST were elevated. The NOAEL for these effects was 125 mg α-tocopheryl acetate/kg body weight (Abdo et al., 1986).

Vrolijk et al. (2015) found that in skin keratinocytes at a concentration of 10 mM vitamin E, the enzyme activity of GST π significantly decreased by 74% (*p* ≤ 0.001). This finding is also likely to occur in other tissues, including the kidney. The inhibition of the protective activity of GST-π could be the cause of tumour-promoting effect of vitamin E in mice (Mitchel and McCann, 1993). El-Hak et al. (2019) studied the effects of vitamin E supplements (daily 500, 1,000, and 2,000 mg vitamin E/kg body weight) in male albino rats. Treatment with higher doses of vitamin E caused significant changes in haematology, biochemistry and histological damage, including the kidneys. In the renal cortex dilatation of the renal tubules and atrophy of the glomerulus already occurred at a dose of 500 mg/kg bw/day. They concluded that oral supplementation of vitamin E in rats for 30 days was not safe for the liver and kidneys and therefore caution is needed when taking high doses of vitamin E.

Hanzawa et al. (2014) investigated the influence of vitamin E on vitamin K metabolism. Rats were fed vitamin K1 or vitamin K2 (menaquinone-4) at levels of 0, 10, 50, or 500 mg/kg for 6 weeks. Vitamin K1 decreased with increasing amounts of vitamin E in various tissues, including the kidney. However, in rats given vitamin K2, vitamin E consumption did not reduce vitamin K2 in kidney tissue. The studies in humans confirmed the findings that high doses of vitamin E affected blood clotting mediated by vitamin K1, while vitamin K2 is not directly involved in blood clotting but regulates the state of the arteries.

In a study by Jansen et al. (2016) mice were given food with different amounts of vitamin E for 6 weeks. Food with the highest vitamin E content contained a dose of 25x RDA (375 mg vitamin E/kg food = 75 mg/kg bw/day). In the kidney, effects of high vitamin E intake were seen in all inflammatory biomarkers in male mice. Statistically significant increases were found for ALT, AST, MCP-1, IL-6, TNF-α, and PAI-1 but not for resistin. In female mice, resistin alone showed a statistically significantly higher concentration, but not in the other renal biomarkers. This study showed that male mice were more sensitive to higher doses of vitamin E. In male mice, an increase in the anti-oxidant status in the kidney was also observed.

The administration of an oral dose of 25x the recommended daily intake of vitamin E in mice showed an increase in several inflammatory parameters in the kidney. Biomarkers of oxidative stress, antioxidant capacity and redox status were not affected, as could be expected from human studies (Singh et al., 2005).

**FIGURE 5:**
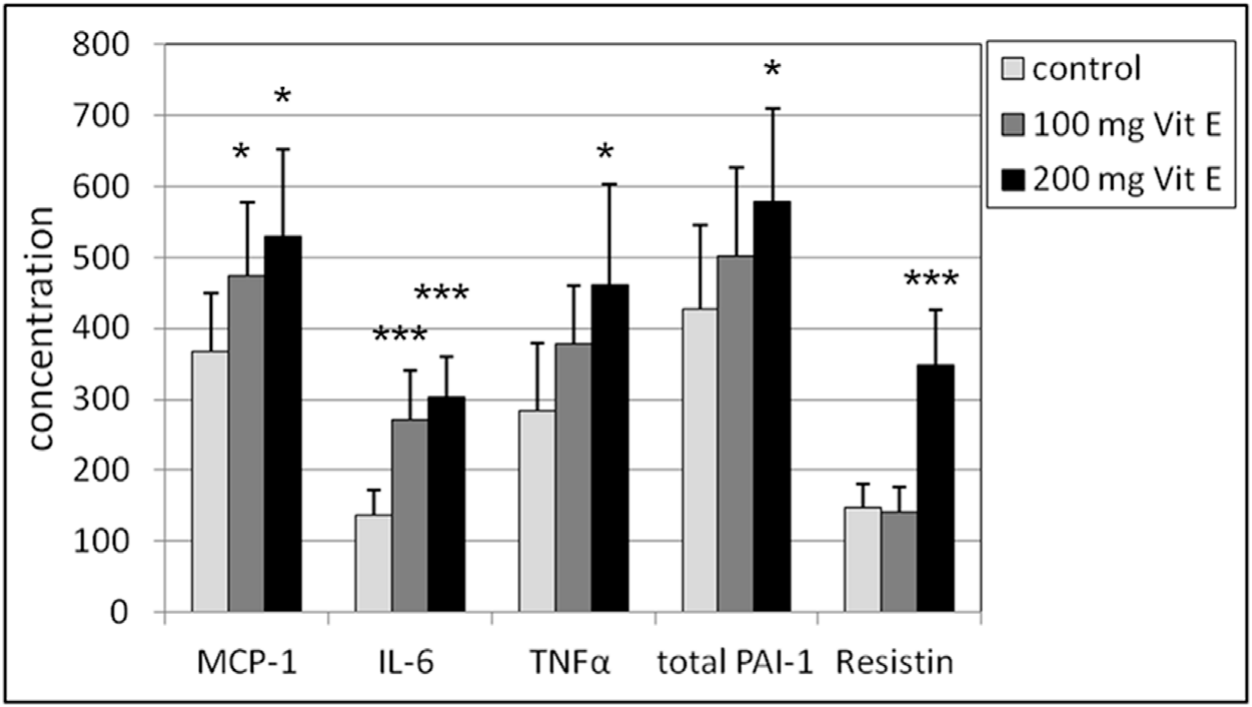
Renal supernatant concentrations in male mice of MCP-1, IL-6, TNF-α, resistin (expressed in pg/mL) and PAI-1 (expressed in μg/mL). Statistics: *p < 0.05 vs. control group; ***p < 0.001 vs. control group. Reprinted from Jansen et al. (2018).

In a short-term study of 14 days (Jansen et al., 2018), male mice were exposed to an intraperitoneal dose of 100 and 200 mg vitamin E/kg body weight/day. Biomarkers of oxidative stress-related processes and biomarkers of tissue toxicity and inflammation were determined. The most adverse effects were observed in the kidney due to a significant increase in the inflammatory biomarkers MCP-1 and IL-6 at a dose of 100 mg/kg bw/day and TNF-α, resistin and PAI-1 were significantly increased at a dose of 100 mg/kg bw/day, 100 mg/kg bw/day, and 200 mg/kg bw/day, respectively (Figure 5). In particular, the dose of 100 mg vitamin E/kg bw/day, which corresponds to approximately 1,100 mg vitamin E/kg bw/day for humans, is close to the upper limit of vitamin E toxicity.

In summary, the possible adverse effects were reported in a number of recent rodent studies in which vitamin E was supplemented, as shown in Table 4. The adverse effects are mainly at the level of biomarkers in blood biochemistry (ALT and AST) or inflammatory biomarkers in kidney tissues (MCP-1, IL-6, TNF-α, PAI-1, and resistin). As explained in the previous chapters, these biomarkers of inflammation can be considered undesirable for the kidney. The question remains whether these biomarkers in rats and mice can be directly extrapolated to the human situation. But measurements in the kidney tissue can only be done in animal studies. It should be noted that a factor of 11.7 should be used in the conversion from mice to humans, so that the adverse effects at the lowest amount of supplementation of vitamin E (Jansen et al., 2016; Jansen et al., 2018) fall within the human range of 878–1,170 mg/day. Potential intakes of vitamin E within this range are lower than the UL of 1,000 mg/day advised in the US.

**TABLE 4:** Summary of rodent studies that reported effects of vitamin E supplementation on the kidneys.

Author	Species	Dose (mg/kg bw)	Overall effect	Disease state	Number of animals	Duration	Exposure	Effect
Trachtman et al. (1996)	Rats, male	6.7	+	IgA nephropathy	30	8 weeks	food	Lower level of MDA
Koya et al. (1997)	Rats, male	40	+	Streptozotocin-induced diabetes	23	10 weeks	intra-peritoneal	Prevention of Glomerular Dysfunction
Hahn et al.(1998)	Rats, male	50	+	Remnant kidney rats	36	2 weeks	Food	Reverse glomerulosclerosis
Jansen et al. (2016)	Mice, male and female	75	-	Normal animals	30	6 weeks	food	Increase of inflammation and cytotoxic biomarkers
Jansen et al. (2018)	Mice, male	100	-	Normal animals	22	14 days	intra-peritoneal	Increase of inflammation biomarkers
El-Hak et al. (2019)	Rats, male	500	-	Normal animals	24	30 days	gavage	Alteration of hematology, biochemistry, histology in the kidney
Hanzawa et al. (2014)	Rats, male	500	-	Normal animals	24	6 weeks	food	Decrease in vitamin K1
Zhao et al. (2018)	Rats, male	1,000	+	Streptozotocin-induced diabetes	28	24 weeks	gavage	Improvement of renal tubular epithelial cells and histology

+, overall positive effects; -, overall negative effects.

In the rodent studies, the findings of adverse effects of vitamin E supplementation on the kidneys are more abundant than in human studies. As shown in Table 4, supplementation of doses higher than 75 mg/kg bw show an adverse effect on the kidneys. Why the study of Zhao et al. (2018) with a high dose of 1,000 mg/kg bw, show completely divergent results compared with all other studies cannot be explained, except that they used streptozotocin-induced diabetic rats. In the study of Koya et al. (1997) streptozotocin-induced diabetic rats were also used but with a rather low dose of vitamin E.

**FIGURE 6:**
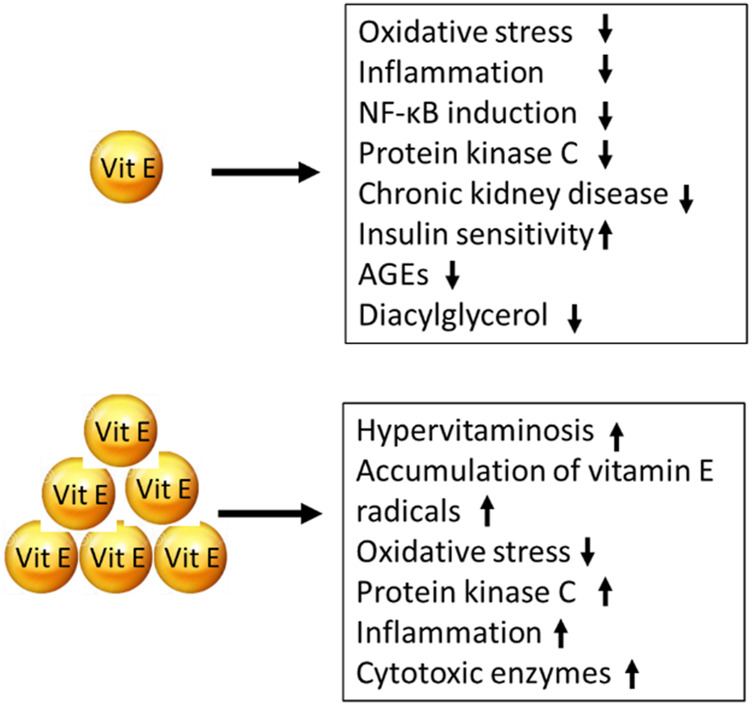
Effects of vitamin E supplementation using low doses (upper part) and high doses (lower part).

The overall effects of vitamin E supplementation both in human and rodents studies are shown schematically in Figure 6.

## 8 Tissue biomarkers of renal toxicity

Several biomarkers were elevated in the kidney after vitamin E supplementation in rodents. They represent inductions that cause adverse and pro-inflammatory effects in the kidney. In this chapter the severity of the elevated biomarkers in the kidney is explained as observed in human studies: ALT and AST (Abdo et al., 1986), MCP-1, IL-6, TNF-a, PAI-1, and resistin (Jansen et al., 2016; Jansen et al., 2018).

The enzyme alanine aminotransferase (ALT) is present in high concentrations in the liver, but is also present in the kidney. Also aspartate aminotransferase (AST) is present in the kidneys. An induction of ALT and a low ratio of AST/ALT are associated with CKD mediated by insulin resistance, in middle-aged women (Ochiai et al., 2020).

Monocyte chemoattractant protein-1 (MCP-1) plays an important role in renal inflammation in both animal models of renal disease and renal patients. In a mice study, Göser et al. (2005) reported that inhibition of MCP-1 significantly reduced progressive renal damage in diabetic nephropathy, which was confirmed by Haller et al. (2016). Boels et al. (2017) also showed that MCP-1 inhibition reduced tissue inflammation in diabetic nephropathy.

Interleukin-6 (IL-6) has been shown to have an impact on CKD (Jones et al., 2015). In a review by Magno et al. (2019) it was concluded that the IL-6 family of cytokines is involved in many renal diseases and may play a deleterious or protective role. In a cohort study of 14,611 patients (Batra et al., 2021), high levels of IL-6 were shown to be associated with a higher risk of serious adverse cardiovascular events in CKD patients. Zhang et al., 2014 showed that tumour necrosis factor-alpha (TNF-α) propagate renal damage in mice. In another study, Awad et al., 2015 showed that the induction of TNF-α in mice plays an important role in the development and pathogenesis of diabetic kidney injury.

Plasminogen activator inhibitor type-1 (PAI-1) has major fibrosis effects in the kidney. Inhibition of PAI-1 may prevent CKD progression and is likely to stimulate its regression. Eddy and Fogo, (2006) and Małgorzewicz et al. (2013) reviewed the literature on PAI-1 in relation to the renal disease. They concluded that elevated PAI-1 levels can lead to a variety of kidney disease.

Serum levels of resistin were markedly elevated in CKD patients with both advanced and mild to moderate renal impairment (Axelsson et al., 2006). Zhang et al. (2015) showed that the levels of serum resistin in the CKD patient group were significantly higher than those in the control group. In addition, they found that resistin levels positively correlated with the duration of dialysis in CKD patients.

In summary, it is convincing that elevated renal-related inflammation biomarkers play an important role in possible adverse effects on the kidney.

## 9 Conversion of UL of vitamin E from human to rodents

For the conversion of the dose of vitamin E from rodents to humans, the calculation by the Food and National Board (FNB) of the UL for humans can be used (Hathcock et al., 2005). They used an uncertainty factor of three from animal to human and a factor of two from sub chronic to chronic exposure. So, the conversion of 1 mg vitamin E/kg bw/day from rodents to humans will lead to a conversion factor of 11.7 from rodents to males of 70 kg bw. Springett et al. (2015) determined on the basis of equivalent relative body surface areas that the conversion factor from rat to human is 10.5 and from mouse to human is 11.7 for a person of 65 kg body weight.

## 10 Effects of vitamin E on renal oxidative stress

Based on the intrinsic properties of vitamin E molecular structure with both lipid and water-soluble part, vitamin E can act as a transfer agent between the lipid-water interface. Vitamin E promotes the strengthening of endogenous antioxidant defences and can protect the pancreas, kidneys, eyes and nervous system in animal models from oxidative stress (Pazdro and Burgess, 2010). But high levels of vitamin E can potentially lead to dangerous effects by a disruption of the oxidative stress detoxification chain (Abudu et al., 2004).

In the HOPE study it was shown that there are no beneficial effects of vitamin E administration (daily dose of vitamin E of 400 IU (268 mg) on renal complications (Lonn et al., 2005). However, it was concluded that the progression of CKD is associated with a higher oxidative stress, with lipid peroxidation more pronounced in haemodialysis patients and protein oxidation in peritoneal dialysis patients. In a study by Tbahriti et al. (2013), patients with CDK of four different severity classes and two dialysis groups (haemodialysis and peritoneal) showed lower antioxidant status and higher oxidative stress with disease severity.

The detoxification of oxygen radicals from long-chain fatty acids to harmless compounds by a sequence of antioxidant species that lowers the oxidation potential in each step is shown in Table 5. This requires a cooperation of numerous compounds. If one compound such as vitamin E is present in excess, the chain will be disturbed and an excess of vitamin E radicals will remain with pro-oxidant properties.

**TABLE 5:** Redox potentials of redox couples that take part in the detoxification chain from radical formation in the lipid phase towards the water phase.

Redox couple	Redox potential (volt)
H_2_O/^**.**^OH	+2.33
H_2_O_2_/^**.**^OH	+0.38
Vitamin E (oxidized/reduced)	+0.37
Coenzyme Q (oxidized/reduced)	+0.10
Vitamin C (oxidized/reduced)	+0.08
Cystine/cysteine	−0.22
Glutathione (oxidized/reduced)	−0.24
Lipoic acid (oxidized/reduced)	−0.29
NAD(P)^+^/NAD(P)H	−0.32

**FIGURE 7:**
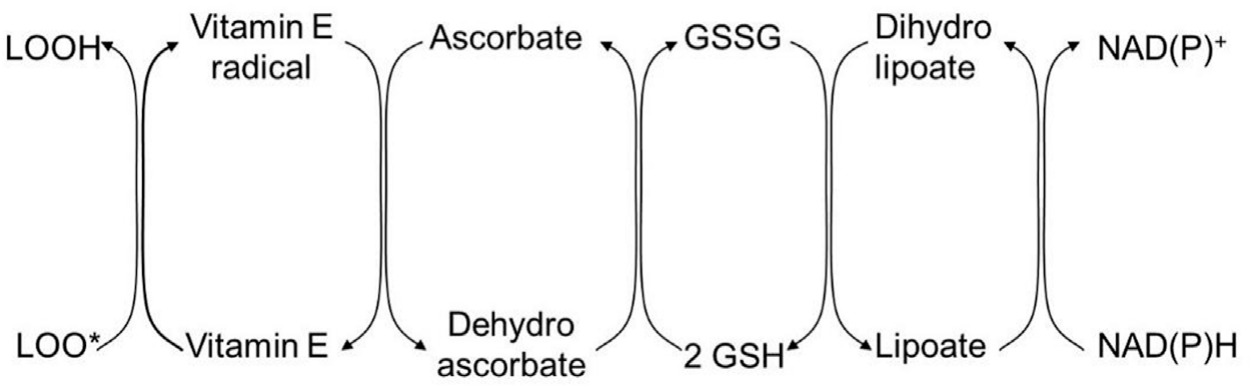
Schematic representation of the detoxification of oxygen radical originated from long-chain polyunsaturated fatty acids in the lipid phase to water-soluble harmless compounds such as NAD(P)H. LOOH, long-chain polyunsaturated fatty acid; LOO, radical of LOOH; GSH, glutathione; GSSG, oxidized glutathione; NAD(P)H, nicotinamide adenine dinucleotide phosphate.

Does vitamin E also have beneficial effects on the kidneys by lowering oxidative stress? The pro-oxidation can be inhibited by cooperation with other antioxidants, such as vitamin C, lipoic acid, glutathione, and NAD(P)H as shown schematically in Figure 7, but only if all oxidants are present at the same level for radical transfer.

Lower levels of vitamin E supplementation can improve the non-enzymatic antioxidant activity through a lower concentration of malondialdehyde and a higher content of glutathione (Bucioli et al., 2011).

In a recent dose-response study of Zhang et al. (2022) in patients with end-stage renal disease, it was found that higher levels of serum vitamin E resulted in a higher anti-oxidant status and a consequently lower oxidative stress as measured by the total reactive oxygen species.

Positive effects of vitamin E were also reported in a review by Bergin et al. (2021). It was concluded from 18 studies that vitamin E supplementation reduces oxidative stress as measured by malondialdehyde in haemodialysis patients. The mean dose was 384 mg/day.

Another pathway for the induction of oxidative stress is the formation of Advanced Glycation End products (AGE) that occurs particular in the kidneys. Accumulation of AGEs in the kidney will decline its function, particularly in patients with diabetes. AGEs are product from the non-enzymatic reaction of the amino groups in proteins, lipids, and nucleic acids with the carbonyl groups of reducing sugars. They can exist as protein-bound AGEs and AGE free adducts, also in food. Examples of these AGE molecules are pyrraline, carboxymethyl lysine, pentosidine, methylglyoxal, deoxyglucosone, and many others.

In conclusion, the most important factor for various renal diseases could thus be the onset and progression of oxidative stress. Vitamin E can influence this process by the low-level intake, which increases its anti-oxidant status. Therefore, the dose level of vitamin E, possibly in combination with other antioxidants, plays an important role in kidney disease.

## 11 Gender differences of vitamin E effects in human and animal models

In some studies, it was found that there is a gender difference in the effects of vitamin E levels. Hara et al. (2021) investigated the relationship between dietary vitamin E intake and renal function in a Japanese population. In this study, a lower intake of tocopherols was found to be associated with lower renal function in Japanese women, while this association was not observed in men. For women, significantly lower renal function was associated with lower levels of β-, γ-, and δ-tocopherol, but not α-tocopherol. In the Japanese men, only α-tocopherol levels were associated with lower renal function.

Jansen et al. (2016) also noted striking differences in renal biomarkers between male and female mice in an additional vitamin E study. In male mice, almost all renal biomarkers of inflammation were statistically significantly elevated (ALT, AST, BAP, MCP-1, IL-6, TNF-α, and PAI-1), while in female mice only one biomarker was elevated (resistin). Therefore, possible sex differences in the effects of therapeutic vitamin E supplementation on renal disease development and progression can be expected.

## 12 Conclusion of the findings of this review

A review of the literature on the safety of oral intake of vitamin E indicates that the toxicity of vitamin E on the kidney is rather low in human studies, even at high doses. However, there are large discrepancies in the effects and the dose-response relationship, even at doses higher than the ULs (Bendich and Machlin, 1988). On the contrary, studies in rodents show adverse effects on the kidneys at much lower doses of vitamin E comparable with doses used in human studies. Therefore, it should be emphasized that high doses of vitamin E that are close to the upper limit of toxicity but still are recommended by manufacturers and considered harmless and beneficial, can potentially cause several adverse effects in the kidneys, also in humans. Higher concentrations of inflammatory biomarkers were observed in the kidney tissue of male mice. This suggests that there may be a gender difference in both vitamin E metabolism and its effect on the development and progression of the kidney disease. Whether these effects are also valid for humans, remains to be investigated. Given the results in some reported rodent studies, the UL for vitamin E may need to be reassessed based on the increased number of renal biomarkers of inflammation.

## 13 Vitamin E supplement recommendations for humans

The antioxidant and anti-inflammatory effects of vitamin E have been well studied. However, the data in the literature regarding the beneficial effects of this vitamin for chronic diseases, including renal disease, are contradictory, which may be caused by the heterogeneity of renal pathology.

When considering the ranges of beneficial and adverse effects, it is difficult to give general advice for both beneficial and the prevention of adverse effects. In human studies there is a range of 24–1,206 mg vitamin E/day with no adverse effects. There is no consensus in the rodent studies also. Positive effects are found at low levels of 6.7, 40 and 50 mg/kg bw and at 1,000 mg/kg bw. At intermediate levels of vitamin E (75–500 mg/kg body weight) there are unfavourable increases in inflammatory biomarkers.

Therefore, no definite advice on the amounts of vitamin E supplementation can be given for healthy individuals nor for patients with kidney disease, based on solid evidence from literature.

As a suggestion, we propose the following protocols. If it is necessary for healthy individuals to increase their vitamin E status, a supplementation of 1x or 2x the recommended daily intake (15–30 mg/day) may be useful in addition to a healthy diet containing vitamin E. For patients with kidney disorders higher levels supplementation with vitamin E can be used depending on the nature and course of the disease. Potential doses of vitamin E can be used in the range of 200–400 mg/day, for limited period of time, possibly in combination with other antioxidants, and under medical supervision.

## 14 Future research questions that remain open

There is still a need for *in vivo* data on the effects of vitamin E in humans that are different from those in rodent studies. The potential cytotoxic effects of vitamin E supplements have raised concerns in the literature and therefore a re-evaluation for a lower UL for vitamin E may be necessary. Furthermore, more knowledge is needed about the interaction of vitamin E with other micronutrients and antioxidants to understand their combined action in human physiology with regard to oxidative stress and inflammation.
